# Transition Metal Chelation Effect in MOF-253 Materials: Guest Molecule Adsorption Dynamics and Proposed Formic Acid Synthesis Investigated by Atomistic Simulations

**DOI:** 10.3390/molecules29133211

**Published:** 2024-07-05

**Authors:** Meng-Chi Hsieh, Wei-Lun Liang, Chun-Chih Chang, Ming-Kang Tsai

**Affiliations:** 1 Intelligent Computing for Sustainable Development Research Center, Department of Chemistry, National Taiwan Normal University, Taipei 11677, Taiwan; 2 Department of Chemical and Materials Engineering, Chinese Culture University, Taipei 11114, Taiwan; 3 Department of Chemistry, Fu-Jen Catholic University, New Taipei City 24205, Taiwan

**Keywords:** metal–organic framework, CO_2_ reduction, atomistic simulation

## Abstract

The dynamic characterization of guest molecules in the metal–organic frameworks (MOFs) can always provide the insightful and inspiring information to facilitate the synthetic design of MOF materials from the bottom-up design of perspective. Herein, we present a series of atomistic molecular dynamics simulation for investigating the bipyridine dicarboxylate (bpydc) linker rotation effect on guest molecule adsorption with and without considering the transition metal (TM) chelation in MOF-253 materials. The simulated PXRD patterns of the various linker orientations present the challenge of distinguishing these structural varieties by the conventional crystalline spectroscopic measurements. The observed short inter-TM stable structure may subsequently lead to the formation of a binuclear TM catalytic site, and a proposed formic acid generation mechanism from CO_2_ and H_2_ is derived based upon the density functional theory calculations for the application of CO_2_ reduction.

## 1. Introduction

Synthesized by the self-assembly of metal secondary building units (SBUs) and organic compounds, metal–organic frameworks (MOFs) have been increasingly studied for application over decades [1]. Attributing to the nature of MOFs, for example, their high surface area, versatility, being easily tunable, etc., MOFs could provide a suitable template for catalysts by using the bared SBUs directly [2,3] or using the cooperated metal nanoparticles or functional groups after modification [4,5,6]. Among these modifications, introducing the concept of metal complexes into the MOFs brings the benefit from both homogeneous and heterogeneous catalysts. It is also possible to make use of the experience accumulated over the past century [7].

Compared to other heterogeneous catalysts binding with metal complexes, MOFs are suited for studying the mechanism by the regular arrangement of building units. In the late 1990s, metal–ligand complexes were introduced as linkers by pre-synthesis. Later, Seo et al. included the racemic Ru(2,2′-bipy)_3_^2+^ into L-POST-1 by post-synthesis modification (PSM) [8]. Lin and coworkers utilized the M[4,4′-(HO_2_C)_2_-bpy]_2_bpy^2+^ with Zn to synthesize the M-doped MOF and further showed the capability of photo-harvest and energy transfer [9]. They also introduced the Ir, Re, and Ru complex to UIO-67 by the mix-and-match synthetic strategy with the pre-synthesized linker [10]. Owing to the vacant chelating sites that provide the possibility of post-synthetic metalation, the linkers containing nitrogen atoms have gained increasing attention in the last decade [11]. Because of the wide application of 2,2′-bipyridine (bpy) due to the photo-related properties of its metal complexes, it is one of the most notable bidentate chelators [12]. In 2010, Bloch et al. replaced the biphenyl dicarboxylate in DUT-5 with 2,2′-bipyridine-5,5′-dicarboxylic acid (bpydc) as the linker to the synthesis of the aluminum-based MOF-253. The adsorption of flue gas showed increasing selectivity to CO_2_ over N_2_ after the PSM strategy introduced Cu(BF_4_)_2_ to MOF-253. The PdCl_2_ was also introduced to MOF-253. Both PdCl_2_ and Cu(BF_4_)_2_ insertion maintained the framework, being observed by the powder X-ray diffraction (PXRD) spectrum [13]. Then, a similar idea was adopted by the post-synthetic exchange (PSE) of the linker in UIO-67 to create UIO-67-bpydc [14]. There are some other MOFs with open bpydc linkers that were designed to achieve specific capabilities [15,16].

Since MOF-253 is a two-dimensional structure, the tube-like channel could provide a clear vision of how the guest molecule approaches the structure. Furthermore, MOF-253 has been investigated for CO_2_ restoration [17] and CO_2_ reduction reaction (CO_2_RR) [18,19,20]. In a previous study, the reaction pathway of formic acid formation by CO_2_RR with MOF-253 and H_2_ was depicted [21]. It also elaborated on how the idea of homogeneous catalytic mechanisms could inspire the reaction in framework structures and how the steric effects of MOFs could affect these reactions. The computational results provided the thermodynamical details of the reaction mechanism, but the dynamical interplay between reactant and metal center remains unsettled. Additionally, the linker rotation is one of the factors that affect the collision between molecules in MOFs, but it was not well explored [22].

**Figure 1:**
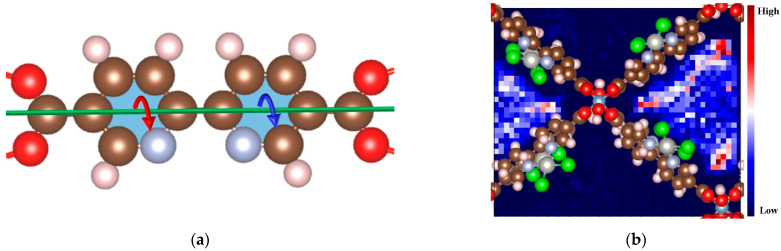
The schematic representations of (**a**) bpydc moiety rotation and (**b**) gas diffusion in MOF-253. The brown, red, pink, indigo, gray, and green denote carbon, oxygen, hydrogen, nitrogen, palladium, and chlorine atoms, respectively. The red and blue curved arrows (**left**) suggest that the two bpydc moieties’ rotations do not necessarily correlate. The color blocks (**right**) denote the probability of guest molecule diffusion projected in a two-dimensional perspective.

The linker rotation can bring new structural diversity and subsequently results new transition metal chelation effect. This structural diversity could consequently introduce various steric hindrance or electronic properties [23]. The rotation originates from the flexibility of organic components, especially for linkers with rigid parts hanging by single bonds. All of the topology [24], the guest molecule [25], and the electronic distribution [26] decide the feasibility of the rotation. 2,2′-bipyridine-5,5′-dicarboxylate, as linkers, confers two rotation portions (Figure 1). Each of them has a C_py_-C_carbonyl_ single bond to connect the terminal carboxyl group, which is fixed on the node, and they have C_py_-C_py_ single bonds to connect with one another. Wu et al. have demonstrated the rotation of bpydc from *cis*-NN (N and N′ in the same direction of the linker) to stable *trans*-NN (N and N′ in a different direction of linker) with 0.42 eV activation energy by quantum computational methods [17]. They concluded that the synthetic processes under room temperature could promote the transformation between the isomers and lead to different lattice parameters.

In this study, the investigation focuses on the contribution of the linker’s rotation and the guest molecule’s distribution in MOF-253 by classical mechanics, molecular dynamics (MD), and density functional theory (DFT) simulation. The simulated PXRD spectra also give insight into the metal-incorporated MOF-253. Herein, an example of the transition metal (TM) dimer formed by the rotation of linkers as the catalyst is discussed. Predictably, the dimer might shortly give a synergetic effect with a good design of the incorporated complexes.

## 2. Results and Discussion

### 2.1. The Dynamics Phenomenon of Linker in MOF-253

In order to understand the underlying thermodynamical difference described at two different theory levels (FF vs. DFT), the rotation barrier along the C1-C2-C3-C4 dihedral angle of the linker with an orientation from trans-NN to cis-NN was calculated using the unit cell model shown in Figure S1. The calculated DFT barrier is approximately predicted at 10.7 kcal/mol in comparison with the corresponding case at 18.4 kcal/mol using FF calculation. The lower energy barrier predicted by DFT appears to indicate that linker rotation could be observed at a T < 700 K condition if the computationally expensive DFT-MD simulations are carried out. However, the FF-MD simulations at the T > 700 K condition can still provide a qualitative thermal-dependent description for the dynamic interplay of guest molecules and linker of MOF materials.

The FF-MD simulations started with using the supercells of L, L_6_, LPdCl_2_, and (LPdCl_2_)_6_ without the presence of gas molecules. The initial linker orientation adopted cis-NN orientation for all types of supercells (Figure S1a,b). All supercells were pre-equilibrated for 0.5 ns, and Table S1 summarized the thermodynamics at different temperatures during the production runs. As shown by the standard deviation of the average energy and temperature, it is reasonable to interpret that these supercells were under the equilibrium condition using the frozen lattice-constant models. Table S2 summarized the histograms of the dihedral angle of C1-C2-C3-N4 based upon the results of L and L_6_ supercells. A linker orientation transition was observed for cis-NN (180 degree) to trans-NN (0 degree) transformation in the case of T > 700 K, and is schematically demonstrated in Figure S2. For the cases at T ≤ 700 K, the bpy moiety could still be well twisted as depicted by the distribution of the C1-C2-C3-N4 angle in Table S2.

The dihedral angle of O5-C6-C7-C8 describes the distortion between the pyridine ring and the carboxylate anchor. For all types of supercells (with and without metal chelation), the pyridine ring can flip in respect to the non-mobile carboxylate anchor at T > 700 K as summarized in Table S3. With the inclusion of PdCl_2_ chelation and the LPdCl_2_ and (LPdCl_2_)_6_ supercells, the dihedral angles of O5-C6-C7-C8 at the resting condition were found to be well twisted to around 150 degrees, suggesting the presence of substantial steric hindrance introduced by the metal chelation. If the O5-C6-C7-C8 flips to zero degrees, a face-to-face PdCl_2_ dimer structure would form, consequently providing a short Pd-Pd distance structure at about 3.48 Å as schematically shown in Figure S3.

As implied by the aforementioned molecular dynamics simulations, the thermal energy at the finite temperature condition could populate the various linker orientations with or without the metal chelation. This structural entropic effect may influence the guest molecules’ adsorption as well as the design strategy of the post-synthesis modification of MOF-253 materials.

### 2.2. The Adsorption of the Guest Molecules in MOF-253

The adsorption behavior of guest molecules, including CO_2_, N_2_, and H_2_, was also investigated using the molecular dynamics simulations. The comparison of the optimized geometries of these molecules using FF and DFT theory levels is listed in Table S4. Tables S5–S7 present the recorded statistics for the CO_2_ in the vacuum, MOF-253, and PdCl_2_-chelated MOF-253 models, respectively, at various temperatures. Tables S8 and S9 report the counterparts of N_2_ and H_2_ in the vacuum, respectively. The adsorption histogram of CO_2_, H_2_, and N_2_ in L and LPdCl_2_ models are schematically presented by the heat maps in Figure S5, in which each color pixel (block) represents the events of guest molecules appearing in the 0.5 × *b*-axis × 0.5 Å^3^ cuboid, and are projected on the two-dimensional map of ac-plane. The color code of each pixel denotes the event count based upon the recorded snapshots during the production run. Figures S6 and S7 provide the similar results of projecting on the ab-plane and bc-plane, respectively, using the exact same procedure as described above. All guest molecules were initially placed in one pore—two separated pores are present in the current simulation supercell. The distribution of color pixels for CO_2_ appears to be most localized, sequentially followed by N_2_ and H_2_, as observed in the L and LPdCl_2_ series of simulations. N_2_ and H_2_ appear to be more mobile based upon their notable color pixels recorded near the central region of the pores. The localization adsorption of CO_2_ in respect to N_2_ simulated under 300 K consistently aligns with the previous experimental observation in MOF-253 and MOF-253-Pd materials, as CO_2_ is a stronger adsorbate than N_2_ [13].

All of the guest molecules have shown a preferential adsorption at nodes situated at the linker moieties with or without the PdCl_2_ chelation. The higher probability of node adsorption may emphasize the importance of node engineering development to introduce the catalytic capability at this region. However, such a design approach could result in more complex synthetic challenges in comparison with the straightforward post-synthesis transition metal chelation. The mobile guest molecule diffusion could still result in substantial adsorption events at the PdCl_2_ site if the pressure of the guest molecule is well controlled.

**Figure 2:**
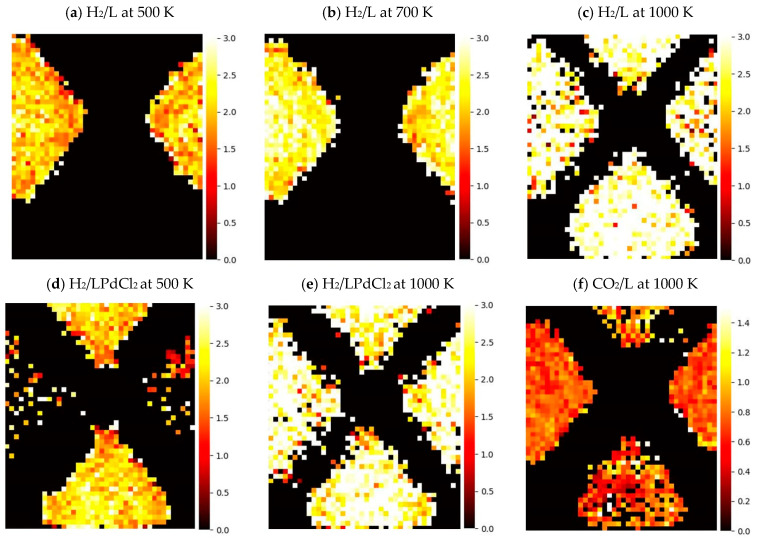
The heat maps of the average speed projected on an *ac*-plane of MOF-253: (**a**–**c**) 1 H_2_ in L at 500, 700, and 1000 K, respectively; (**d**,**e**) 1 H_2_ in LPdCl_2_ at 500 and 1000 K, respectively; (**f**) 1 CO_2_ in L at 1000 K. Each color pixel of the heat map denotes the recorded molecular speed observed in the volume 0.5 × *b*-axis × 0.5 (Å^3^) and weighted by the number of observed events. The unit of *y*-axis denotes the average speed at Å/100 fs. Figure S1 provides the schematic perspective of the *ac*-plane of MOF-253.

Figure 2 provides the statistics of the observed dynamics of guest molecules (H_2_ and CO_2_) penetrating the MOF flamework to enter the neighboring pore. One hydrogen molecule was initially residing at one pore and was physically blocked by the linker orientation of the minimum energy structure. Without the PdCl_2_ chelation, this small guest molecule appears to localize at the initial pore at simulations of 500 K and 700 K, until reaching the temperature at 1000 K to penetrate between the neighboring pores (see the comparison of Figure 2a–c). Darker pixels were generally observed near the linker surface region in comparison with the center region of the pores, suggesting the presence of the intermolecular “friction” that resulted from the interactions of the MOF-253 framework. With the incorporation of PdCl_2_ chelation, H_2_ appears to significantly populate at both pores at 500 K and 1000 K (see Figure 2d,e). The steric hindrance introduced by PdCl_2_ chelation can twist the neighboring linkers, as schematically shown in Figure S1b—the presence of PdCl_2_ moiety can break the coplanar nature of bipyridine. This consequently facilitates the PdCl_2_-coordinated linker rotation as being notably observed at 500 K.

### 2.3. The Examination of the PXRD Simulation

**Figure 3:**
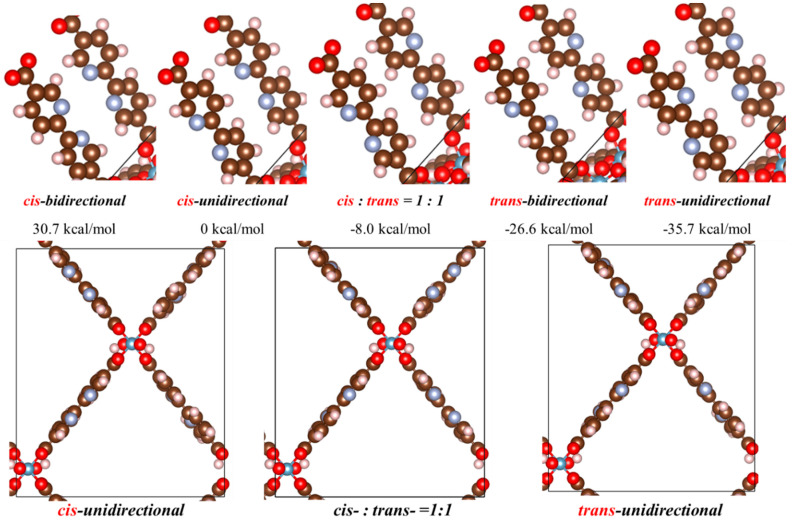
The schematics of linkers and the relative DFT-optimized energy to the cis-unidirectional in kcal/mol of MOF-253 isomers. Side views of the optimized structures shown below. The brown, red, pink, and indigo denote carbon, oxygen, hydrogen, and nitrogen atoms, respectively.

In order to understand the relative energetics of the orientations of the linkers in MOF-253, we conducted DFT simulations to take into account the possible minimum structures using the current supercell model and identified five stable geometries as listed in Figure 3. The lowest energy orientation is found to be the trans-unidirectional orientation, being substantially lower in energy at 35.7 kcal/mol than its cis-unidirectional counterpart. The cis-unidirectional orientation is believed to be more favorable for the multi-dentate metal chelation, and also was reported in the previous post-synthesized experimental modification [13]. Both of the low-energy geometries (trans-unidirectional and trans-bidirectional) could not provide multi-dentate binding sites for post-synthesis metal chelation, where the least-stable cis-bidirectional case (66.4 kcal/mol higher than the lowest-energy orientation) may provide a tetra-dentate binding site if the metal moiety is large enough to form the inter-linker coordination bonds.

**Figure 4:**
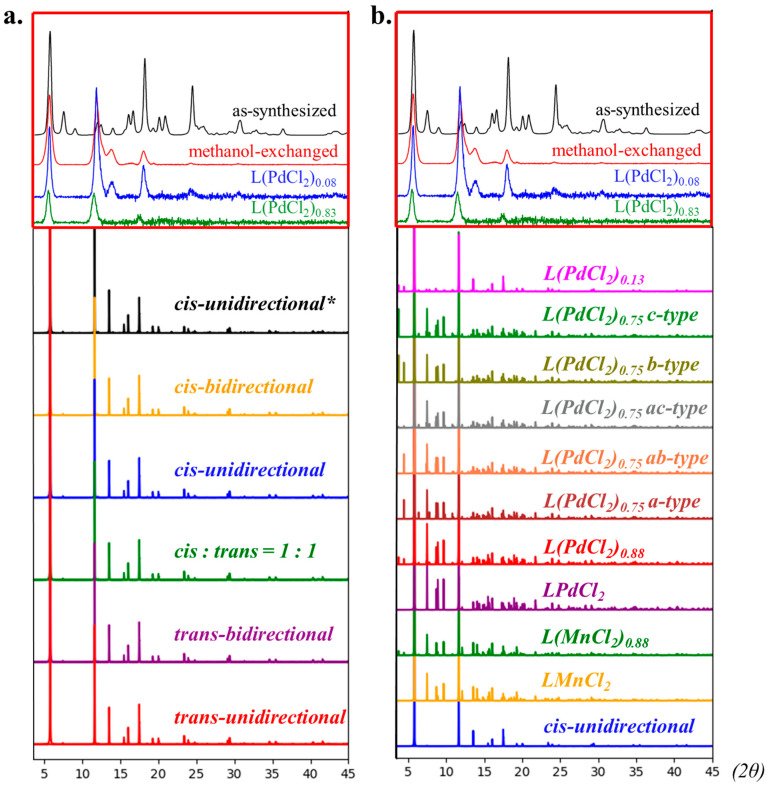
The simulated PXRD spectra. The red squares in (**a**,**b**) are experimental PXRD spectra that were retrieved from the report by Bloch et al. [13]. (**a**) The comparison between different isomers of L. The black line with the label * is the L model that was built from L(PdCl_2_) and then had the metal complexes “vertically” removed. It converted to the PXRD data without re-optimization by DFT computation. (**b**) The comparison between different numbers of MCl_2_ incorporated into the *cis*-NN structures that were discussed in our previous study [21].

Figure 4a summarizes the calculated PXRD spectrum using these five optimized DFT structures, i.e., the hypothetical pristine 1 × 1 × 1 supercells using VESTA [27]. All of these five linker orientations appear to have visually identical PXRD patterns so that the presence of diverse linker orientations may not be experimentally distinguishable by the spectroscopic feature of PXRD. However, it should be noted that the cis-unidirectional* model takes into account the linker distortion effect that resulted from the MCl_2_ chelation, and the consequent PXRD spectrum is calculated upon the vertical removal of the MCl_2_ fragment while maintaining linker distortion. Such a comparison between the cis-unidirectional and cis-unidirectional* cases only give a trivially deviated intensity of a 2θ angle at around 12 and 17 degrees, and consequently reinforces the issue of identifying linker distortion using PXRD spectra. Figure 4b summarizes the models by considering the various percentages of MCl_2_ chelation in which the current supercell can maximumly accommodate eight MCl_2_ bindings. Substantial spectroscopic features are observed at the small-angle region (<10 degree) as well as some notable features between 13 and 17 degrees based upon the comparison between simulated MCl_2_-chelated and non-chelation models. Such a theoretical observation is consistent with the PXRD spectrum of as-synthesized MOF-253 [13], particularly for the <10 degree region. However, the observed spectroscopic feature between 13 and 17 degrees could either have resulted from the cases of MCl_2_ chelation or non-chelation, consequently re-emphasizing the importance of the determination of the small-angle feature at <10 degree.

### 2.4. The Catalytic Mechanism for Another LPdCl_2_ Isomer

**Figure 5:**
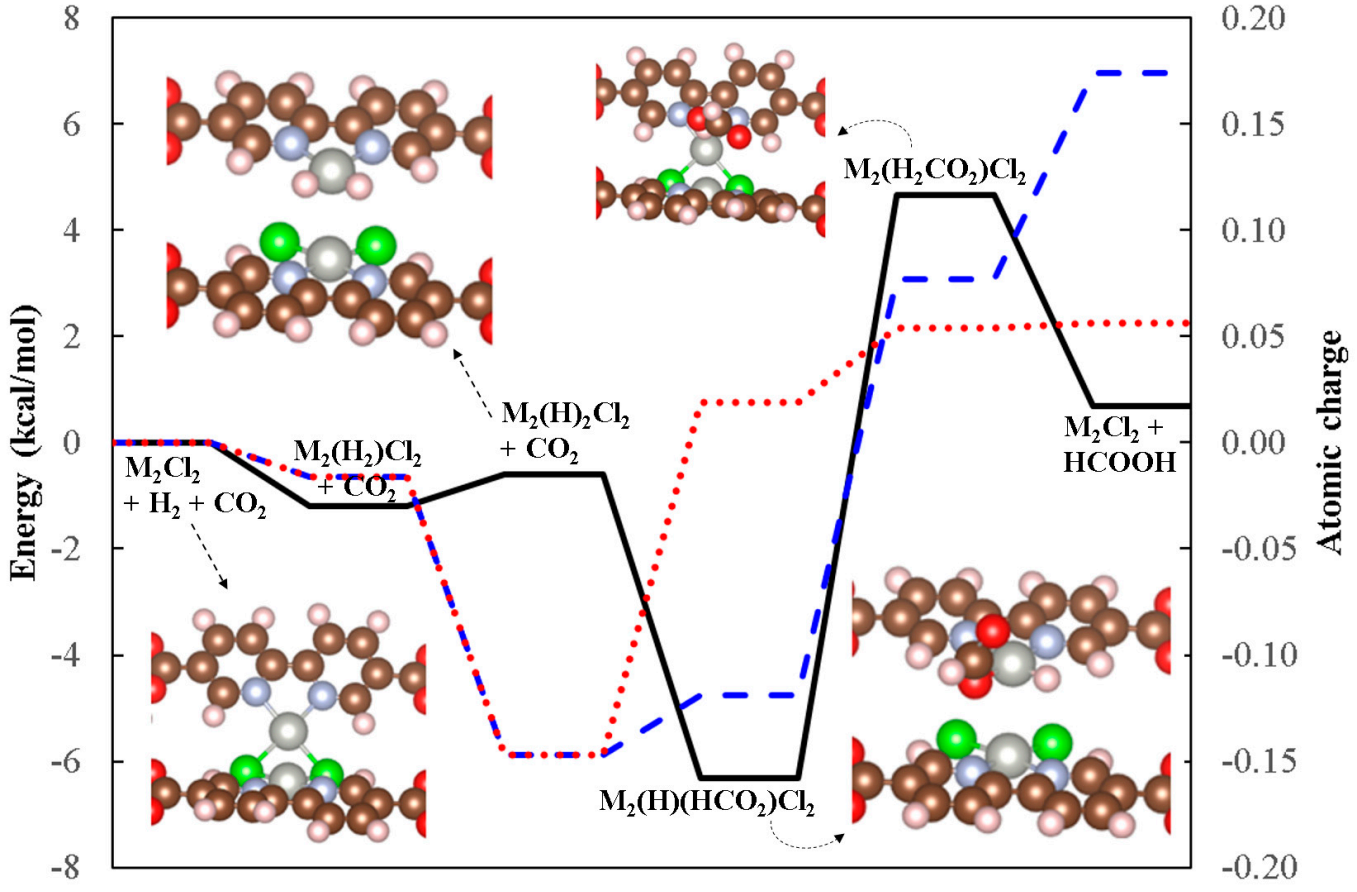
The energetic profile (black line) and atomic charge of hydrogen along the formic-acid-generation reaction coordinate on Pd_2_Cl_2_ catalytic site. The red dotted and blue dashed lines denote the calculated Hersheld atomic charges of H bound to O and C, respectively.

We reported the theoretical analysis for the formic acid generation from CO_2_ and H_2_ by metal–chloro chelation in MOF-253 materials, in which PdCl_2_ chelation was computationally recognized as one of the accessible catalytic candidates for the aforementioned CO_2_RR catalysis [21]. The interplay between the neighboring PdCl_2_ chelation moieties is investigated in this study, and several interacting PdCl_2_ models from the steric hindrance perspective are theoretically optimized, as schematically shown in Figure S8. The cis-bidirectional structure (see Figure S8c) is identified to be marginally higher in energy than the lowest-energy cis-unidirectional case (see Figure S8a) by 3.14 kcal/mol. This minimal energetic difference indeed suggests that the cis-bidirectional orientation could be highly populated during the post-synthesis modification process. Additionally, the Pd-Pd distance of the cis-bidirectional model is calculated as 3.362 Å, which may facilitate the reductive elimination of two chloro-ligands to form a dimeric Pd_2_Cl_2_ moiety, as schematically depicted in Figure 5. The corresponding reaction energy for cis-bidirectional 2×PdCl_2_@MOF-253 to Pd_2_Cl_2_@MOF-253 + Cl_2_(g) is predicted to be 72.55 kcal/mol endothermically, which could be accessible at elevated temperature conditions and is favorable by the entropic effect. The resultant binuclear Pd(I) moiety could convert CO_2_ and H_2_ to formic acid as schematically shown in Figure 5.

Hydrogen can be exothermically adsorbed on this binuclear site with the adsorption energy at −1.91 kcal/mol, followed by inter-hydrogen bond breaking to form a PdCl_2_(H)_2_Pd moiety with a minimally endothermic reaction energy at +0.58 kcal/mol. The incoming CO_2_ can easily access the hydrido-ligand to form *OCHO intermediate through gaseous molecule diffusion, which can eventually result in the formation of formic acid with the predicted barrier at less than 11 kcal/mol. The desorption energy of formic acid is estimated to be only 3.97 kcal/mol exothermically due to the bidentate character of HCOOH coordination elongating the metal–chloro bridge bond. The desorption of HCOOH can finally restore the binuclear site back to its acting catalytic condition as the Pd_2_Cl_2_ moiety. The proposed binuclear catalytic site shows highly accessible energetics for converting CO_2_ and H_2_ to formic acid. A similar binuclear framework chelated in MOF-253-like materials has recently been synthesized by Wang et al. for C-H bond activation applications [28].

## 3. Methodology

The MOF-253 materials were described by the periodic boundary condition model using the orthorhombic unit cell (*a* = 23.59 Å, *b* = 6.91 Å, *c* = 19.84 Å) where the lattice constants remained frozen in the calculations. The atomic coordinates within the supercell were fully optimized, adapting the same supercell construction procedure [21], using density functional theory (DFT) or force field (FF) levels of theory. All calculations were carried out with the Amsterdam Modeling Suite (AMS) program [29,30]. There were various types of supercells used in this study, i.e., L denoting the 1 × 1 × 1 bare MOF-253 unit cell without metal chelation and LPdCl_2_ denoting eight units of PdCl_2_ coordinated in L. Additionally, L_6_ denoted six replicas along the *b*-axis as the 1 × 6 × 1 supercell, and (LPdCl_2_)_6_ denoted the corresponding case containing 48 PdCl_2_ chelation moieties.

UFF4MOF was adopted for the FF calculations [31], i.e., the parameter set derived from UFF [32] but extended to MOFs and TM complexes. Notably, the development of UFF4MOF was calibrated for MIL-53 materials, in which it contains the same SBUs as MOF-253. FF-MD simulations were carried out for understanding the guest molecule distribution at the finite temperature conditions (300–1000 Kelvin) under the NVT ensemble with the Berendsen thermostat. The integration timestep of FF-MD was chosen at 0.5 fs. The cutoff of the non-bonding interaction was chosen at 15 Å while particle smash Ewald summation was used for the Coulomb interaction. Despite the decomposition of MOF-253 at 700 K and the metal coordinated case at 650 K [13] observed via the experimental thermal gravimetric analysis, the temperature of the current MD simulations still exceeded beyond 700 K during the exploration of the dynamic phenomena of linker movements. As explained in the above Results and Discussion Section, the linker rotation barrier is substantially overestimated at FF level than the predicted barrier at DFT. Conducting FF-MD simulations at temperatures higher than 700 K can still provide the chemical significance for understanding the atomistic dynamics at a highly mobile condition while maintaining reasonable computational costs. The FF-MD simulations were pre-equilibrated for 0.5 ns, followed by the production run for another 0.5 ns with snapshots recorded every 100 fs.

DFT simulations were conducted with the generalized gradient approximation (GGA) with the Perdew–Burke–Ernzerh (PBE) exchange-correlation functional [33] with all of the atoms described by a triple-zeta-quality atom-centered basis set with one polarization function. For the inclusion of van der Waals interactions, the D3 version of Grimme’s dispersion Becke–Johnson damping [34,35] was applied. Moreover, the scalar relativity effect was treated by the zero-order regular approximation (ZORA) approach [36]. The convergence criteria were set as 10^–5^ eV for total energy change and 0.01 Å for a total Cartesian step change. The DFT calculation was carried out by the BAND module of AMS. Additionally, the PXRD pattern was simulated by the Powder Diffraction Pattern utility in VESTA software.

## 4. Conclusions

In this study, we present a series of classical molecular dynamics simulations for understanding the linker dynamic effect on the guest molecule adsorption in MOF-253 materials. All of the possible linker orientations were explored and subsequently optimized by DFT calculation, and the simulated PXRD patterns were investigated. The motion of bpydc rotation was found to facilitate the guest molecule penetrating various pores in MOF-253, and the steric hindrance introduced by TM chelation can even benefit the linker rotation. The corresponding simulated PXRD patterns of these non-TM-chelated structures indicate the challenges of identifying distinguishable spectroscopic features for classifying linker orientations. The spectroscopic features at 2θ angle less than 10 degrees appear to suggest the presence of TM chelation. Interestingly, a PdCl_2_-chelated minimum structure, labeled as *cis*-bidirectional orientation, contains the short Pd-Pd distance that can result in the formation of a binuclear Pd_2_Cl_2_ catalytic site. Such a binuclear reaction center was found to substantially favor the formic acid generation mechanism from CO_2_ + H_2_ with the predicted catalytic barrier at less than 11 kcal/mol based upon the DFT calculation results.

## Data Availability

The data are contained within the article.

## Supplementary Materials

The following supporting information can be downloaded at: https://www.mdpi.com/article/10.3390/molecules29133211/s1, Figure S1: The schematic representation of the MOD-253 model; Figures S2–S4: Linker rotation dynamics; Figures S5–S7: Heat maps of MD simulation with guest molecule adsorption; Figure S8: Schematic representation of TM chelation and the energetics; Tables S1–S3: Statistics of the structure dynamics of MD simulations; Table S4: Guest molecule structure comparison; Tables S5–S9: Statistics of MD simulation with gas adsorption.
