# Supplementary material for: Transition Metal Chelation Effect in MOF-253 Materials: Guest Molecule Adsorption Dynamics and Proposed Formic Acid Synthesis Investigated by Atomistic Simulations
Source: Molecules. 2024 Jul 5;29(13):3211. doi: 10.3390/molecules29133211 (PMC11243041; doi:10.3390/molecules29133211)
Supplement: Supplementary file 1 [file molecules-29-03211-s001.zip › molecules-3039064-supplementary.pdf]

Transition Metal Chelation Effect in MOF-253 Materials: Guest Molecule Adsorption Dynamics and Proposed Formic Acid Synthesis Investigated by Atomistic Simulations

Meng-Chi Hsieh [1], Wei-Lun Liang [1], Chun-Chih Chang [2,*] and Ming-Kang Tsai [1,3,*]

1 Department of Chemistry and Intelligent Computing for Sustainable Development Research Center, National Taiwan Normal University, Taipei 11677, Taiwan
2 Department of Chemical and Materials Engineering, Chinese Culture University, Taipei 11114, Taiwan
3 Department of Chemistry, Fu-Jen Catholic University, New Taipei City 24205, Taiwan
* Correspondence: zjz8@ulive.pccu.edu.tw (C.-C.C.); mktsai@ntnu.edu.tw (M.-K.T.)

Supporting Information

Table S1. The examination of stability for the MD in different conditions. T_{set} denotes the set temperature for each model. $E_{average}$ and $T_{average}$ denote the average of energy (Hartree) and temperature (K) after 0.5 ns. $E_{average}$* and $T_{average}$* denote the average of energy (kcal/mol) and temperature (K) per 100 picoseconds minus $E_{average}$ and $T_{average}$ relatively. E_{std} and T_{std} denote the standard deviation of energy (kcal/mol) and temperature (K) per 100 picoseconds. L and $LPdCl_2$ are the PBC models with the unit cell parameters (a = 23.59 Å, b = 6.91 Å, c = 19.84 Å). L_6 and $(LPdCl_2)_6$ are the extended PBC models that elongated 6 times along the b-axis.

		**L**				**L_6**				**$LPdCl_2$**				**$(LPdCl_2)_6$**			
**T_{set}**		**300**	**500**	**700**	**1000**	**300**	**500**	**700**	**1000**	**300**	**500**	**700**	**1000**	**300**	**500**	**700**	**1000**
**$E_{average}$ (Hartree)**	**0.5-1.0ns**	-1.5901	-1.1707	-0.7542	-0.1440	-9.5154	-7.0241	-4.5256	-0.8122	-1.7806	-1.3184	-0.8548	-0.1556	-10.6833	-7.9106	-5.1302	-0.9290
**$E_{average}$* (kcal/mol)**	**0.5-0.6ns**	-0.1242	-0.0049	0.0891	1.6708	-0.0797	0.0949	-0.3016	12.3114	0.0838	-0.2258	0.5923	0.8751	-0.0006	-0.2427	0.4037	-2.5099
	**0.6-0.7ns**	0.0069	-0.0871	-0.3910	1.8118	-0.1909	0.4449	0.3157	3.1200	0.0764	0.0266	-0.1829	1.2110	-0.1017	-0.0760	-0.2256	-5.8419
	**0.7-0.8ns**	0.1532	0.3303	0.2221	2.3244	-0.2526	-0.1887	0.1172	-5.3625	-0.1356	0.1108	0.0165	0.5062	-0.1401	-0.2635	0.4160	1.7934
	**0.8-0.9ns**	-0.0063	-0.2108	0.1235	-2.0856	0.2380	-0.0342	-0.6949	-4.5401	-0.0417	-0.0098	-0.0303	-0.9417	0.0709	0.3549	-0.7395	1.2166
	**0.9-1.0ns**	-0.0296	-0.0275	-0.0437	-3.7214	0.2852	-0.3170	0.5636	-5.5288	0.0171	0.0981	-0.3956	-1.6506	0.1715	0.2273	0.1454	5.3418
**$T_{average}$ (K)**	**0.5-1.0ns**	298.2476	499.9363	700.2932	999.1544	300.0150	500.1503	699.9011	999.6388	300.3667	500.2108	700.1913	999.6245	299.9636	499.9000	699.9377	999.8672
**$T_{average}$* (K)**	**0.5-0.6ns**	-0.0338	0.1363	0.3766	0.4289	-0.0058	0.1461	-0.0970	-0.1086	-0.0579	-0.2205	0.3206	0.3002	-0.0704	0.2363	0.6854	-0.5115
	**0.6-0.7ns**	-0.2411	0.4637	0.4636	-1.1578	0.0067	-0.1719	-0.0017	0.0533	0.4317	0.7307	-0.1713	0.3349	-0.1668	0.2677	-0.8554	0.2288
	**0.7-0.8ns**	0.1778	-0.3918	-0.7283	-0.3016	-0.0186	0.1359	0.2319	0.3622	-0.7924	-1.5400	0.4617	-0.8633	-0.1194	0.0593	0.0147	-0.2343
	**0.8-0.9ns**	-0.0676	0.5925	0.2790	0.6409	0.0265	-0.1122	-0.2657	-0.0900	0.4558	0.0026	-0.6137	-0.5577	-0.0292	-0.2199	0.4255	0.0263
	**0.9-1.0ns**	0.1647	-0.8007	-0.3909	0.3895	-0.0088	0.0021	0.1326	-0.2169	-0.0372	1.0272	0.0027	0.7860	0.3858	-0.3434	-0.2702	0.4907
**E_{std} (kcal/mol)**	**0.5-1.0ns**	0.3124	0.5336	0.7766	2.8899	0.7699	1.2750	1.9055	7.8453	0.3277	0.5814	1.0282	3.8197	0.8036	1.3651	2.2816	7.0155
	**0.5-0.6ns**	0.2930	0.5223	0.7490	1.2789	0.7556	1.2960	1.8151	4.5564	0.2871	0.5877	1.3561	2.2170	0.7801	1.4028	2.6449	7.3557
	**0.6-0.7ns**	0.3272	0.5036	0.7529	1.2361	0.7125	1.3287	1.7167	3.2865	0.3795	0.5580	0.9289	2.0832	0.8014	1.3187	2.0852	5.4183
	**0.7-0.8ns**	0.2883	0.4858	0.7920	1.1735	0.6993	1.2093	1.8581	3.9484	0.3108	0.5861	0.8790	1.6008	0.7746	1.2958	2.3193	5.9035
	**0.8-0.9ns**	0.2924	0.4936	0.7374	1.9887	0.7635	1.1996	1.7287	3.4430	0.3003	0.5770	0.8695	3.6436	0.8031	1.4007	2.0906	4.8629
	**0.9-1.0ns**	0.2949	0.5066	0.7012	1.8948	0.7555	1.2014	2.1168	2.7388	0.3016	0.5333	0.7188	6.4660	0.8194	1.2905	2.0000	5.5308
**T_{std} (K)**	**0.5-1.0ns**	11.6967	19.7935	27.4847	38.8613	4.8138	7.9996	11.2679	15.9404	11.2039	19.1483	25.9403	37.4139	4.6130	7.5630	10.6778	15.4082
	**0.5-0.6ns**	11.3395	20.0405	26.8182	38.7800	4.8040	8.1300	10.7987	16.2853	11.1449	18.8332	26.9987	37.1621	4.5709	7.3094	10.4702	15.4602
	**0.6-0.7ns**	11.7580	19.4851	27.3250	39.3423	4.9346	7.7098	11.4123	15.7024	11.3688	19.1884	25.4466	37.4133	4.5462	7.4973	10.4409	15.0935
	**0.7-0.8ns**	11.7555	19.3993	27.4450	38.8161	4.8445	7.8881	11.4117	15.7735	11.0807	18.9222	25.3061	38.2332	4.7207	7.3281	10.6349	15.3066
	**0.8-0.9ns**	12.2734	20.0108	28.2295	37.6742	4.6049	7.9469	11.3543	15.6334	11.4158	18.5244	26.2128	37.0495	4.5379	7.6330	11.0200	15.3479
	**0.9-1.0ns**	11.3502	20.0261	27.6215	39.7133	4.8840	8.3204	11.3660	16.3190	10.9794	20.1659	25.7390	37.2493	4.6742	8.0205	10.7653	15.8343

Table S2. The summary information of the dihedral angle of $C_{①}$-$C_{②}$-$C_{③}$-$N_{④}$ in different conditions after 0.5ns. The $C_{①}$-$C_{②}$-$C_{③}$-$N_{④}$ shows the relative rotation of connected pyridine at the linker. The column Average denotes the average value of the dihedral angle from 0.5ns to 1.0ns. The column 100fs shows the dihedral angle at the initial 100fs, indicating the starting structure. The column Max and Min indicate the maximum and minimum of the dihedral angle from 0.5ns to 1.0ns relatively.

		Average	100fs	Max	Min
L	300	180.2972	180.0553	219.6115	142.1108
	500	179.1531	178.7169	230.2746	123.9684
	700	179.2241	178.7169	239.9251	112.7674
	1000	180.0938	179.9467	359.9880	0.0156
L_6	300	179.4884	179.1408	220.3670	143.2998
	500	180.5568	180.3101	226.0148	133.4352
	700	180.5018	180.3101	235.8864	121.1484
	1000	179.4848	180.3101	359.9941	0.0100

Table S3. The summary information of the dihedral angle of $O_{⑤}$-$C_{⑥}$-$C_{⑦}$-$C_{⑧}$ in different conditions after 0.5ns. The $O_{⑤}$-$C_{⑥}$-$C_{⑦}$-$C_{⑧}$ shows the direction of the N atom in one of the pyridines. The column Average denotes the average value of the dihedral angle from 0.5ns to 1.0ns. The column 100fs shows the dihedral angle at the initial 100fs, indicating the starting structure. The column Max and Min indicate the maximum and minimum of the dihedral angle from 0.5ns to 1.0ns relatively.

		Average	100fs	Max	Min
L	700	181.1302	181.3825	236.3196	125.7233
	1000	188.4163	180.3404	359.9798	0.0016
L_6	700	181.2295	181.5358	267.1335	117.1086
	1000	177.6271	179.8277	359.9920	0.0073
$LPdCl_2$	300	151.6990	152.6107	175.2802	128.3870
	500	151.2232	152.6107	179.9024	120.2098
	700	150.3864	152.6107	181.3703	113.9661
	1000	44.8919	152.6107	359.8728	0.0422
$(LPdCl_2)_6$	300	156.6318	156.7821	178.2184	137.2417
	500	153.5883	155.1016	180.2827	123.6429
	700	152.6709	154.8705	188.6247	119.6932
	1000	221.6794	207.6905	359.6820	0.7492

Table S4. The comparison between the bond length of molecules between UFF and BAND results. The diff(F to L) was denoted as the difference that was calculated by ｜1-(F/L)｜x100%.

	**UFF**	**BAND**	**diff (UFFtoBAND,%)**
**N_2**	1.12	1.11	1.24
**H_2**	0.71	0.75	5.72
**CO_2**	1.20	1.18	2.18

Table S5. The statistics of two CO_2 molecules in the empty PBC cells (V = 1×2×1 and 2V = 1×4×1) using the experimental MOF-253 lattice constants. T_{set} denotes the set temperature for each model. $E_{average}$ and $T_{average}$ denote the average of energy (Hartree) and temperature (K) after 0.5 ns. $E_{average}$* and $T_{average}$* denote the average of energy (kcal/mol) and temperature (K) per 100 picoseconds minus $E_{average}$ and $T_{average}$ relatively. E_{std} and T_{std} denote the standard deviation of energy (kcal/mol) and temperature (K) per 100 picoseconds.

		2 CO_2 in V				2 CO_2 in 2V			
T_{set}		**300**	**500**	**700**	**1000**	**300**	**500**	**700**	**1000**
$E_{average}$ (Hartree)	**0.5-1.0ns**	0.0088	0.0146	0.0204	0.0293	0.0087	0.0146	0.0208	0.0296
$E_{average}$* (kcal/mol)	**0.5-0.6ns**	0.0609	0.0582	0.0224	0.0866	0.0326	0.0521	0.1143	0.0128
	**0.6-0.7ns**	0.0203	0.0092	0.0208	0.0072	0.0081	0.0260	0.0557	-0.0135
	**0.7-0.8ns**	-0.0085	-0.0202	-0.0052	-0.0923	-0.0061	-0.0051	0.0317	0.1007
	**0.8-0.9ns**	-0.0402	-0.0346	-0.0117	-0.0170	-0.0151	-0.0202	-0.0751	-0.0117
	**0.9-1.0ns**	-0.0325	-0.0127	-0.0263	0.0155	-0.0196	-0.0529	-0.1266	-0.0884
$T_{average}$ (K)	**0.5-1.0ns**	300.0449	500.0624	699.8476	1000.203	300.0060	499.9947	699.9980	999.9347
$T_{average}$* (K)	**0.5-0.6ns**	-0.0306	0.2110	-0.1186	0.1484	-0.0029	0.0050	-0.1492	0.5297
	**0.6-0.7ns**	0.0896	0.0308	-0.5988	0.1485	-0.0520	-0.0954	0.0061	-0.4530
	**0.7-0.8ns**	0.0292	-0.0080	0.4691	-0.3262	0.0916	0.0563	0.2235	0.1861
	**0.8-0.9ns**	-0.0084	0.0411	0.1157	0.3345	0.0041	0.0692	-0.0839	-0.0684
	**0.9-1.0ns**	-0.0799	-0.2749	0.1327	-0.3053	-0.0408	-0.0350	0.0036	-0.1945
E_{std} (kcal/mol)	**0.5-1.0ns**	0.0564	0.0447	0.0413	0.0868	0.0348	0.0447	0.0954	0.0840
	**0.5-0.6ns**	0.0484	0.0421	0.0407	0.0771	0.0231	0.0293	0.0368	0.0573
	**0.6-0.7ns**	0.0355	0.0233	0.0339	0.0557	0.0254	0.0239	0.0420	0.0879
	**0.7-0.8ns**	0.0399	0.0266	0.0372	0.0340	0.0265	0.0260	0.0429	0.0526
	**0.8-0.9ns**	0.0554	0.0302	0.0401	0.0729	0.0334	0.0191	0.0351	0.0433
	**0.9-1.0ns**	0.0280	0.0288	0.0309	0.0745	0.0357	0.0302	0.0218	0.0346
T_{std} (K)	**0.5-1.0ns**	6.6254	9.8168	14.6748	16.0028	4.9690	7.7509	12.5807	16.5237
	**0.5-0.6ns**	9.0272	8.6736	15.6065	16.8311	3.6840	9.9320	18.6644	11.3563
	**0.6-0.7ns**	5.8786	6.2982	20.9900	10.4150	7.7079	8.3076	12.1744	21.1069
	**0.7-0.8ns**	6.1043	7.0350	9.0297	14.4702	4.7866	6.6186	10.0279	14.8955
	**0.8-0.9ns**	6.2316	9.5616	14.0989	13.5906	3.6033	4.8095	11.1592	16.2014
	**0.9-1.0ns**	5.2436	15.0431	10.6081	22.2546	3.8287	8.1243	8.3821	17.5177

Table S6. The examination of stability for the MD of CO_2 addition to L with different conditions. T_{set} denotes the set temperature for each model. $E_{average}$ and $T_{average}$ denote the average of energy (Hartree) and temperature (K) after 0.5 ns. $E_{average}$* and $T_{average}$* denote the average of energy (kcal/mol) and temperature (K) per 100 picoseconds minus $E_{average}$ and $T_{average}$ relatively. E_{std} and T_{std} denote the standard deviation of energy (kcal/mol) and temperature (K) per 100 ps. L is the PBC model with the unit cell parameters (a = 23.59 Å, b = 6.91 Å, c = 19.84 Å). L_6 is the extended PBC model that elongated 6 times along the b-axis.

		CO_2 in L				2 CO_2 in L (same pore)				2 CO_2 in L (different pores)				CO_2 in L_6			
T_{set}		300	500	700	1000	300	500	700	1000	300	500	700	1000	300	500	700	1000
$E_{average}$ (Hartree)	**0.5-1.0ns**	-1.5850	-1.1643	-0.7434	-0.1165	-1.5844	-1.1588	-0.7322	-0.1058	-1.5848	-1.1588	-0.7324	-0.1055	-9.5151	-7.0178	-4.5174	-0.7979
$E_{average}$* (kcal/mol)	**0.5-0.6ns**	0.1105	0.4059	-0.2117	1.2673	0.0241	0.2189	0.2158	1.8094	-0.1306	0.2159	-0.2101	0.9968	0.0289	0.2505	0.1646	7.5682
	**0.6-0.7ns**	0.0918	-0.3487	0.0512	-0.2007	0.0957	0.1871	0.2564	3.2510	-0.6055	0.0764	-0.8103	0.3614	0.0902	-0.1766	0.1220	2.6330
	**0.7-0.8ns**	-0.2010	-0.0082	-0.5166	-0.7271	0.1612	0.2356	-0.3435	-2.2353	0.2479	0.4194	0.2423	1.9376	-0.0708	0.1364	0.4217	-1.7261
	**0.8-0.9ns**	0.1885	-0.2654	0.2686	0.5083	0.0143	-0.0462	-0.0968	-0.6680	0.6611	0.1312	0.5507	-1.5590	-0.4549	0.5451	-0.0067	-5.3777
	**0.9-1.0ns**	-0.1897	0.2164	0.4085	-0.8479	-0.2954	-0.5953	-0.0318	-2.1571	-0.1729	-0.8429	0.2274	-1.7368	0.4065	-0.7553	-0.7016	-3.0974
$T_{average}$ (K)	**0.5-1.0ns**	300.2086	499.9689	699.3675	1000.511	299.9550	499.6197	700.1173	999.7213	300.0131	499.8252	699.8065	1000.369	299.9944	499.9180	700.1738	1000.066
$T_{average}$* (K)	**0.5-0.6ns**	0.0751	-0.0584	0.4497	0.4034	-0.2858	-0.3080	0.1980	-0.0042	0.3491	0.2914	0.7674	-0.7712	0.1766	-0.1789	-0.1240	0.8143
	**0.6-0.7ns**	0.2355	0.9266	-0.0747	-0.2570	0.2955	0.8127	0.1170	-0.0893	-0.1564	-1.1748	-0.8896	0.9292	0.0775	-0.1708	0.3878	-0.3414
	**0.7-0.8ns**	-0.4341	-0.3294	-0.5707	1.0535	-0.0558	-0.8237	0.0764	0.9089	0.3476	0.1954	-0.5574	-1.5772	-0.0810	-0.1759	-0.4381	0.2270
	**0.8-0.9ns**	0.3078	-0.7806	0.4576	0.3647	0.2742	0.7564	-0.3388	-1.5762	-0.2604	0.4876	0.1333	0.5817	0.0500	0.4665	-0.0753	-0.3667
	**0.9-1.0ns**	-0.1842	0.2419	-0.2619	-1.5647	-0.2281	-0.4374	-0.0526	0.7609	-0.2799	0.2004	0.5463	0.8375	-0.2231	0.0590	0.2496	-0.3332
E_{std} (kcal/mol)	**0.5-1.0ns**	0.7154	1.1091	1.6076	2.6905	0.8081	1.5936	2.1769	3.6625	0.9625	1.4936	2.1268	4.0294	1.0029	1.6383	2.4342	6.7452
	**0.5-0.6ns**	0.5935	1.0253	1.8008	2.0509	0.7604	1.3659	1.4698	3.4335	0.8138	1.2518	2.4080	2.9383	0.9064	1.5966	1.8732	4.7207
	**0.6-0.7ns**	0.7512	1.0007	1.4544	2.4832	0.8456	1.3604	1.9544	2.4354	0.7498	1.4319	2.0467	3.1909	1.0250	1.5784	2.3119	4.6028
	**0.7-0.8ns**	0.6066	1.1491	1.7688	2.6638	0.7227	1.7498	2.4468	3.4344	0.7818	1.1116	2.0853	4.9934	0.8982	1.7323	2.6961	5.6452
	**0.8-0.9ns**	0.6895	1.2265	1.3225	2.6154	0.8393	1.5033	2.5985	2.6891	0.9190	1.4285	2.0969	3.8329	0.9904	1.3439	2.4233	5.4937
	**0.9-1.0ns**	0.8175	0.9348	1.4639	2.9593	0.7899	1.7818	2.1804	2.5325	1.0211	1.8201	1.6689	3.5311	0.9934	1.6131	2.6349	4.0422
T_{std} (K)	**0.5-1.0ns**	11.8044	19.6093	27.2370	39.5651	11.5570	19.3131	26.8375	39.0883	11.5635	19.2329	27.8184	38.5412	4.7962	7.9856	11.1804	16.2625
	**0.5-0.6ns**	11.8934	19.6860	27.2490	39.6060	11.2814	18.4839	27.1813	40.2876	11.9746	19.1350	27.3248	39.2097	4.5975	7.9224	10.9750	15.6635
	**0.6-0.7ns**	11.6781	19.5314	27.3843	38.7271	11.3834	18.4164	26.4104	39.4349	11.3709	19.1731	28.2579	38.4544	4.7828	8.1987	11.2196	16.3590
	**0.7-0.8ns**	11.9773	19.6479	28.0390	40.1692	12.0348	20.0011	27.7152	38.2536	11.2798	20.0443	28.0156	38.7518	4.7803	8.0008	11.0203	16.5484
	**0.8-0.9ns**	11.8259	19.6522	27.2959	39.4708	11.5718	19.8044	26.3675	39.1283	11.8265	18.8245	27.7422	37.9765	4.9959	7.9207	11.1613	16.6998
	**0.9-1.0ns**	11.6515	19.5262	26.2251	39.8671	11.5090	19.7788	26.5379	38.3292	11.3539	18.9557	27.7626	38.3148	4.8158	7.8776	11.5207	16.0195

Table S7. The examination of stability for the MD of CO_2 addition to $LPdCl_2$ with different conditions. T_{set} denotes the set temperature for each model. $E_{average}$ and $T_{average}$ denote the average of energy (Hartree) and temperature (K) after 0.5 ns. $E_{average}$* and $T_{average}$* denote the average of energy (kcal/mol) and temperature (K) per 100 picoseconds minus $E_{average}$ and $T_{average}$ relatively. E_{std} and T_{std} denote the standard deviation of energy (kcal/mol) and temperature (K) per 100 picoseconds. L_6 is the PBC model with the unit cell parameters (a = 23.59 Å, b = 6.91 Å, c = 19.84 Å). $(LPdCl_2)_6$ is the extended PBC model that elongated 6 times along the b-axis.

		CO_2 in $LPdCl_2$				2 CO_2 in $LPdCl_2$ (same pore)				2 CO_2 in $LPdCl_2$ (different pores)				CO_2 in $(LPdCl_2)_6$			
**T_{set}**		**300**	**500**	**700**	**1000**	**300**	**500**	**700**	**1000**	**300**	**500**	**700**	**1000**	**300**	**500**	**700**	**1000**
**$E_{average}$ (Hartree)**	**0.5-1.0ns**	-1.7813	-1.3130	-0.8441	-0.1409	-1.7818	-1.3081	-0.8350	-0.1229	-1.7816	-1.3079	-0.8340	-0.1173	-10.6849	-7.9047	-5.1197	-0.9252
**$E_{average}$* (kcal/mol)**	**0.5-0.6ns**	0.2694	0.1343	-0.4540	0.5841	-0.2930	0.0419	-0.1394	-1.3875	0.1011	0.1410	0.2591	-2.1137	0.0054	0.1352	-0.3930	-2.0539
	**0.6-0.7ns**	0.0181	-0.4585	1.9519	3.9028	0.3926	-0.2154	0.5596	-0.2957	-0.1371	-0.0688	-0.6604	-1.3781	-0.4266	0.1001	0.8249	0.7668
	**0.7-0.8ns**	0.1900	0.0177	-0.2815	1.6577	-0.5058	0.3319	0.0989	-1.9270	-0.0815	0.4932	0.0633	-0.0745	-0.1727	-0.1728	0.1066	0.4459
	**0.8-0.9ns**	-0.1195	0.2237	-0.1628	-3.1409	0.1634	-0.3921	-0.1765	0.6558	0.3964	-0.6257	-0.4390	3.8434	0.1465	0.3951	-0.0524	-1.1871
	**0.9-1.0ns**	-0.3581	0.0828	-1.0536	-3.0037	0.2430	0.2337	-0.3427	2.9544	-0.2789	0.0603	0.7770	-0.2770	0.4475	-0.4576	-0.4861	2.0284
**$T_{average}$ (K)**	**0.5-1.0ns**	300.3008	500.1973	700.0924	999.6466	300.0337	500.2617	699.7790	1000.154	299.6336	499.6105	699.8281	1000.542	300.1978	500.0694	699.9901	1000.027
**$T_{average}$* (K)**	**0.5-0.6ns**	-0.3500	-0.4718	-0.4524	-1.0896	0.0516	-0.7621	-0.5679	0.6625	-0.8578	-0.4460	1.0167	0.6576	-0.0510	-0.2001	-0.2235	0.5116
	**0.6-0.7ns**	0.4209	-0.6640	-0.9729	1.1690	-0.3314	-0.4600	0.3806	1.1638	0.3825	-0.4003	-0.1909	1.8461	0.0910	0.0475	0.0536	-0.4182
	**0.7-0.8ns**	0.1811	0.2355	0.5919	0.7205	0.3950	0.3538	0.8983	1.1510	0.3502	0.2571	-0.0239	0.1661	-0.1089	-0.1330	0.6803	-0.0095
	**0.8-0.9ns**	-0.2550	1.4050	1.3701	-0.5135	-0.2106	1.2120	-0.5233	-2.5845	0.3041	0.9231	0.1194	-1.7977	0.1445	0.3519	-0.0843	0.4956
	**0.9-1.0ns**	0.0030	-0.5048	-0.5367	-0.2865	0.0954	-0.3436	-0.1876	-0.3929	-0.1790	-0.3339	-0.9213	-0.8721	-0.0755	-0.0664	-0.4261	-0.5795
**E_{std} (kcal/mol)**	**0.5-1.0ns**	0.7416	1.0427	2.1967	5.6119	1.0203	1.4793	1.9961	3.9893	1.0039	1.5420	1.7230	4.0659	1.0319	1.6788	2.6513	5.1546
	**0.5-0.6ns**	0.6340	1.0466	1.5969	2.6941	0.8101	1.6184	1.9878	4.0502	0.9375	1.3153	1.4498	2.9723	1.1393	1.8671	2.6682	4.5125
	**0.6-0.7ns**	0.6610	0.9752	3.1334	4.6793	1.1905	1.4007	1.5564	2.4338	0.9673	1.4679	1.9356	3.6928	1.0249	1.6860	2.7965	5.5468
	**0.7-0.8ns**	0.9210	0.8892	1.3659	6.9989	0.7349	1.2201	2.4383	2.8490	0.8357	1.6005	1.6908	3.3271	0.8887	1.5537	2.3834	5.2856
	**0.8-0.9ns**	0.7528	1.1059	1.3333	5.3433	0.9501	1.7934	1.6373	2.8177	1.0814	1.3384	1.6467	3.6316	0.8906	1.5885	2.7537	4.0879
	**0.9-1.0ns**	0.4962	1.0464	1.7023	3.6962	1.0540	1.1390	2.1087	5.1333	1.0439	1.7312	1.4601	3.8408	0.9814	1.5532	2.4260	5.1615
**T_{std} (K)**	**0.5-1.0ns**	11.2308	18.6915	26.0404	36.8786	10.8306	18.5678	25.7960	36.6344	10.9877	18.3446	26.2868	37.0320	4.6102	7.6675	10.6511	15.3000
	**0.5-0.6ns**	11.1947	18.8725	26.8810	36.8094	11.2820	18.9848	26.0123	37.8124	11.0458	17.7148	26.0461	36.9310	4.6257	7.7118	10.6478	15.4750
	**0.6-0.7ns**	11.4024	18.7849	26.3294	36.4392	10.8143	18.1925	26.1727	35.6222	11.3065	18.7402	26.3187	37.3694	4.5782	7.8892	10.2703	15.1211
	**0.7-0.8ns**	11.2787	18.7291	26.0422	36.6957	10.8536	18.0602	25.5391	36.6600	10.9195	17.9718	26.8350	36.5233	4.5066	7.6517	10.6049	15.9432
	**0.8-0.9ns**	11.2329	17.7342	25.5333	36.9850	10.5585	18.9184	26.5325	36.3952	10.9482	19.4615	25.4823	36.8033	4.7393	7.3623	10.6952	15.0966
	**0.9-1.0ns**	11.0469	19.2607	25.3691	37.4830	10.6370	18.6337	24.7057	36.5859	10.6794	17.7716	26.7445	37.4920	4.6020	7.7161	11.0125	14.8374

Table S8. The examination of stability for the MD of N_2 addition to the cell with different conditions. T_{set} denotes the set temperature for each model. $E_{average}$ and $T_{average}$ denote the average of energy (Hartree) and temperature (K) after 0.5 ns. $E_{average}$* and $T_{average}$* denote the average of energy (kcal/mol) and temperature (K) per 100 picoseconds minus $E_{average}$ and $T_{average}$ relatively. E_{std} and T_{std} denote the standard deviation of energy (kcal/mol) and temperature (K) per 100 picoseconds. V means an empty cell with unit cell parameters (a = 23.59 Å, b = 6.91 Å, c = 19.84 Å). L and $LPdCl_2$ are the PBC models with the same unit cell parameters.

		2 N_2 in V				N_2 in L				N_2 in $LPdCl_2$			
T_{set}		**300**	**500**	**700**	**1000**	**300**	**500**	**700**	**1000**	**300**	**500**	**700**	**1000**
$E_{average}$ (Hartree)	**0.5-1.0ns**	0.0057	0.0096	0.0134	0.0191	-1.5855	-1.1673	-0.7504	-0.1271	-1.7805	-1.3153	-0.8494	-0.1506
$E_{average}$* (kcal/mol)	**0.5-0.6ns**	0.0060	0.0173	0.0282	0.0140	-0.1278	-0.0243	0.8482	1.1020	-0.1048	-0.0554	-0.6870	1.6624
	**0.6-0.7ns**	0.0032	0.0087	0.0031	0.0003	-0.3116	-0.1963	0.0983	2.1060	-0.1627	0.2912	-0.2781	1.0875
	**0.7-0.8ns**	-0.0011	0.0046	-0.0006	0.0019	0.0152	-0.1797	0.8478	-0.1644	0.1388	-0.4378	-0.1018	-0.0923
	**0.8-0.9ns**	-0.0039	-0.0148	-0.0139	-0.0085	0.2589	0.1799	-0.4577	-1.3210	-0.0981	0.0339	0.3364	-0.5454
	**0.9-1.0ns**	-0.0043	-0.0159	-0.0167	-0.0077	0.1652	0.2205	-1.3367	-1.7226	0.2268	0.1682	0.7304	-2.1121
$T_{average}$ (K)	**0.5-1.0ns**	299.9647	499.9314	699.8711	999.9817	300.2913	499.7818	700.1019	999.7102	299.8590	500.7032	699.7734	999.7048
$T_{average}$* (K)	**0.5-0.6ns**	-0.0400	-0.0156	-0.2747	0.3196	0.1557	0.6981	0.2381	-0.6987	0.2020	-0.5504	-0.0054	-1.6974
	**0.6-0.7ns**	-0.0445	0.2179	0.1319	-0.3698	-0.1229	-0.6083	0.1864	0.1847	0.0206	0.2429	-0.9805	-0.6051
	**0.7-0.8ns**	0.1328	-0.4066	0.0107	0.7648	-0.2800	-0.9308	0.9883	0.5388	-0.7473	0.2320	0.9267	1.3251
	**0.8-0.9ns**	-0.0626	0.0629	-0.1425	-0.2638	-0.2277	0.2634	-0.2717	-1.5251	0.1018	-0.7337	-0.1079	0.5639
	**0.9-1.0ns**	0.0143	0.1412	0.2745	-0.4508	0.4748	0.5777	-1.1410	1.5002	0.4230	0.8092	0.1670	0.4135
E_{std} (kcal/mol)	**0.5-1.0ns**	0.0187	0.0246	0.0252	0.0299	0.6876	1.1058	1.8111	2.7766	0.6480	1.2473	1.8268	2.7488
	**0.5-0.6ns**	0.0183	0.0176	0.0197	0.0216	0.6315	0.9508	1.6152	2.2537	0.6944	1.2633	2.1384	2.0881
	**0.6-0.7ns**	0.0150	0.0211	0.0183	0.0298	0.6657	1.0590	1.2230	2.3639	0.5979	1.2252	1.5938	2.7675
	**0.7-0.8ns**	0.0137	0.0167	0.0146	0.0264	0.7145	1.4632	1.0603	2.4636	0.6116	1.2370	1.7706	2.2958
	**0.8-0.9ns**	0.0206	0.0301	0.0195	0.0300	0.7268	0.6623	1.3654	2.2700	0.6404	1.3674	1.8453	2.0975
	**0.9-1.0ns**	0.0222	0.0142	0.0241	0.0346	0.5263	1.1666	2.4233	2.5078	0.5997	0.9894	1.3557	2.7197
T_{std} (K)	**0.5-1.0ns**	5.8214	9.7580	15.4422	17.8596	11.7214	19.1512	27.3797	38.5507	11.2234	18.8617	26.4840	37.1821
	**0.5-0.6ns**	5.7959	10.9060	17.5698	7.2543	11.6994	19.1968	26.9034	39.5266	11.2767	18.5056	26.0899	37.9301
	**0.6-0.7ns**	7.0524	7.5542	7.3708	20.9725	11.9545	18.6595	26.3847	38.4514	11.4888	19.2700	26.7521	37.2170
	**0.7-0.8ns**	3.8549	15.6886	9.3625	7.3093	11.5148	19.0830	28.7220	37.3265	11.1507	18.2521	26.4149	37.1993
	**0.8-0.9ns**	7.7066	5.0594	20.0273	18.1374	11.8067	19.2316	27.8481	38.2633	11.1003	18.7448	26.5489	35.5912
	**0.9-1.0ns**	3.4604	5.3390	18.4744	26.8354	11.6335	19.5568	26.9888	39.1552	11.0828	19.5024	26.6271	37.9245

Table S9. The examination of stability for the MD of H_2 addition to the cell with different conditions. T_{set} denotes the set temperature for each model. $E_{average}$ and $T_{average}$ denote the average of energy (Hartree) and temperature (K) after 0.5 ns. $E_{average}$* and $T_{average}$* denote the average of energy (kcal/mol) and temperature (K) per 100 picoseconds minus $E_{average}$ and $T_{average}$ relatively. E_{std} and T_{std} denote the standard deviation of energy (kcal/mol) and temperature (K) per 100 picoseconds. V means an empty cell with unit cell parameters (a = 23.59 Å, b = 6.91 Å, c = 19.84 Å). L and $LPdCl_2$ are the PBC models with the same unit cell parameters.

		**2 H_2 in V**				**H_2 in L**				**H_2 in $LPdCl_2$**			
**T_{set}**		**300**	**500**	**700**	**1000**	**300**	**500**	**700**	**1000**	**300**	**500**	**700**	**1000**
**$E_{average}$ (Hartree)**	**0.5-1.0ns**	0.0057	0.0096	0.0136	0.0195	-1.5839	-1.1658	-0.7471	-0.1233	-1.7785	-1.3141	-0.8481	-0.1661
**$E_{average}$* (kcal/mol)**	**0.5-0.6ns**	-0.0069	0.0193	-0.0268	0.1349	-0.2906	-0.1970	-0.4896	2.5600	0.1479	-0.2554	0.1763	-2.4232
	**0.6-0.7ns**	-0.0068	0.0219	-0.0651	0.1274	0.3250	-0.5135	0.2072	1.2337	-0.1175	0.1444	-0.7750	-0.4272
	**0.7-0.8ns**	-0.0031	-0.0146	-0.0359	0.0645	0.0408	0.4612	-0.0786	-0.3492	0.0302	0.3635	0.1869	0.9121
	**0.8-0.9ns**	0.0111	-0.0211	0.1210	-0.2174	0.0297	0.0486	-0.4119	-1.5297	-0.0298	0.0067	0.1364	3.0458
	**0.9-1.0ns**	0.0056	-0.0055	0.0067	-0.1094	-0.1048	0.2007	0.7729	-1.9148	-0.0308	-0.2592	0.2755	-1.1075
**$T_{average}$ (K)**	**0.5-1.0ns**	299.9863	500.0416	699.8376	1000.183	299.9034	499.9716	699.4599	1000.029	299.9021	499.8197	699.9100	999.1044
**$T_{average}$* (K)**	**0.5-0.6ns**	0.0834	-0.0259	0.2667	-0.1479	0.0839	0.0469	0.8990	-1.8133	0.3017	-0.0152	-0.3642	1.9483
	**0.6-0.7ns**	-0.2493	-0.0109	0.0301	-0.2217	-0.6602	-0.3309	0.0273	0.8405	-0.0683	1.2370	-1.9576	-1.8752
	**0.7-0.8ns**	0.0928	-0.0583	-0.6270	0.0925	0.0143	-0.3051	-0.7283	-0.4604	0.0350	-1.4343	1.6275	-0.4929
	**0.8-0.9ns**	-0.0058	-0.0150	0.0339	0.0760	0.2574	0.0336	0.5168	0.7625	-0.3754	0.2014	1.0259	-0.2200
	**0.9-1.0ns**	0.0789	0.1100	0.2964	0.2011	0.3045	0.5556	-0.7147	0.6708	0.1070	0.0111	-0.3316	0.6398
**E_{std} (kcal/mol)**	**0.5-1.0ns**	0.0101	0.0223	0.0768	0.1473	0.5801	1.0449	1.6088	3.5455	0.6306	1.0843	1.7409	4.1121
	**0.5-0.6ns**	0.0052	0.0184	0.0201	0.0103	0.6782	0.9061	2.1406	2.0709	0.5309	0.9744	1.3740	3.2189
	**0.6-0.7ns**	0.0048	0.0127	0.0149	0.0071	0.4640	0.9531	1.4436	3.2382	0.7465	1.0361	1.8985	4.0969
	**0.7-0.8ns**	0.0121	0.0153	0.0560	0.0548	0.5883	0.9514	1.2393	3.6034	0.5153	1.0063	1.4996	3.0216
	**0.8-0.9ns**	0.0048	0.0084	0.0335	0.0651	0.4879	1.1454	1.5400	3.3013	0.7385	1.2716	1.8250	4.1170
	**0.9-1.0ns**	0.0057	0.0117	0.0608	0.0570	0.4696	0.9794	1.1493	3.1666	0.5489	0.9722	1.8256	3.7394
**T_{std} (K)**	**0.5-1.0ns**	3.4372	4.3468	10.9561	6.3779	11.9188	19.7345	27.4044	39.4111	11.0970	18.3717	26.4438	37.4561
	**0.5-0.6ns**	1.9878	5.7706	5.8143	4.0538	11.6276	19.8304	27.6662	39.7700	10.8103	18.7531	26.9464	36.7149
	**0.6-0.7ns**	6.7935	3.6354	5.1758	3.5116	11.7331	19.6476	26.9133	39.4486	10.6669	17.9899	27.0725	37.4232
	**0.7-0.8ns**	1.9990	4.0997	18.2493	3.4298	11.9224	19.7041	28.1224	40.3401	11.4965	18.7682	26.6108	36.9803
	**0.8-0.9ns**	1.5671	5.0967	12.0518	7.5924	12.1155	19.3003	27.3060	38.8418	10.8240	17.9818	26.0792	38.5477
	**0.9-1.0ns**	1.5745	2.2880	7.8249	10.2593	12.1843	20.2057	27.0123	38.6433	11.6614	18.2871	25.3794	37.5560

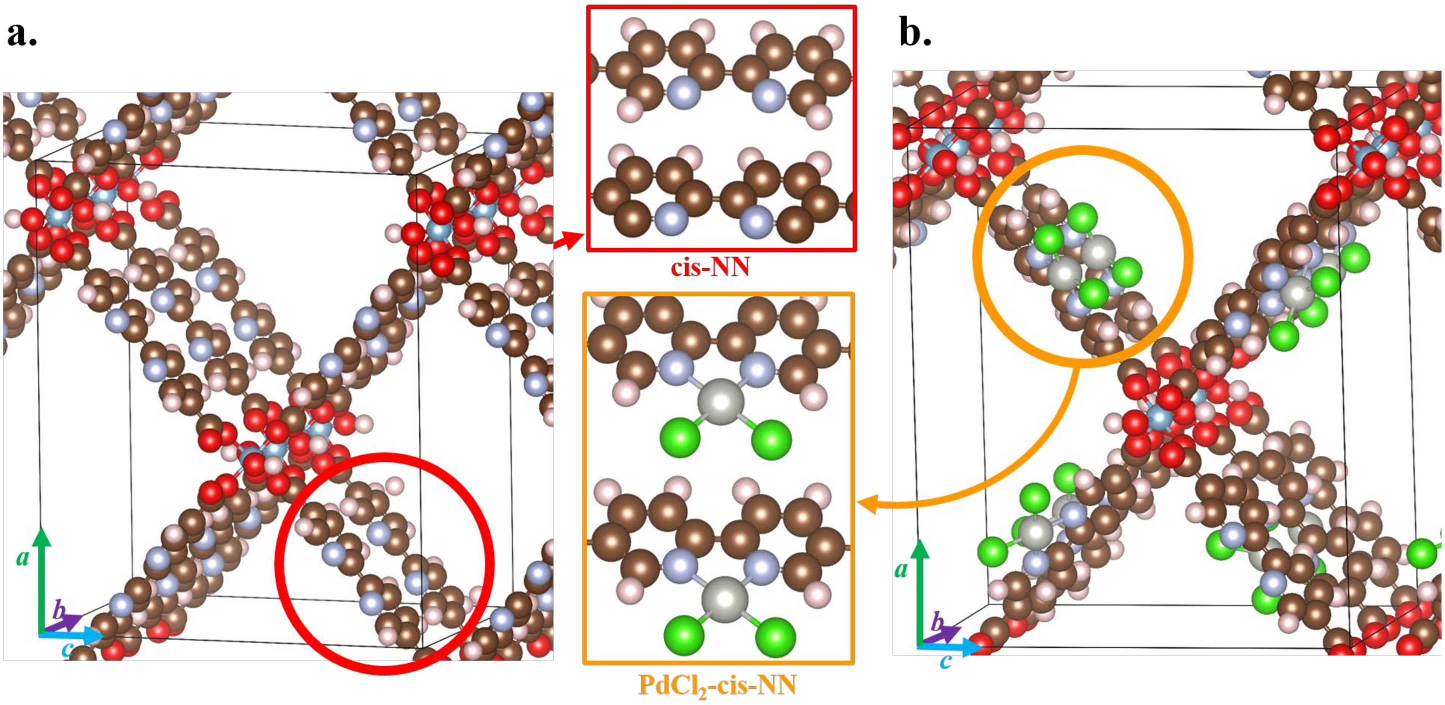

Figure S1. The optimized PBC models of bared L (a) and $LPdCl_2$ (b). The red circle zooms into the linkers of L in the cis-NN form. The orange circle points out the linkers with $PdCl_2$ chelated on the cis-NN coordinate site, denoted as $PdCl_2$-cis-NN.

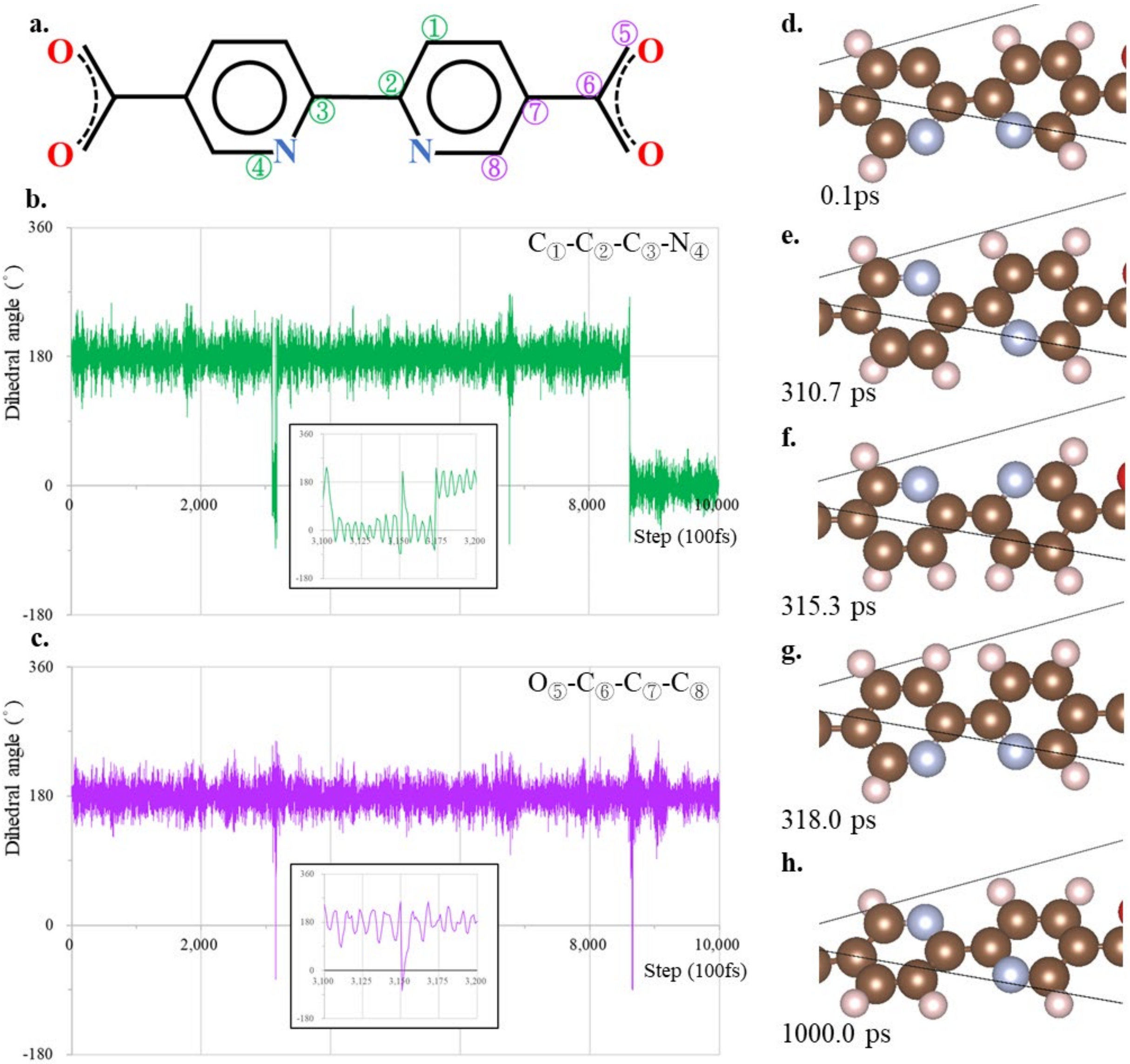

Figure S2. (a) The label shows the definition of dihedral angles, C①-C②-C③-N④ (green) and O⑤-C⑥-C⑦-C⑧ (purple). (b) The green line shows the change of C①-C②-C③-N④ of L from step 0 to 10000 at 1000K (100fs per step). The inner plot is the enlargement of the range from 0.31ns to 0.32ns. (c) The purple line shows the change of O⑤-C⑥-C⑦-C⑧ of L from step 0 to 10000 at 1000K. The inner plot is the enlargement of the range from 0.31ns to 0.32ns. (d)-(h) The structures show the direction difference of pyridine. (d) Both pyridines at the initial structure (step 0) are in the same direction with C①-C②-C③-N④ close to 180° and O⑤-C⑥-C⑦-C⑧ close 180°. (e) The pyridines at 310.7ps (step 3107) are in different directions (trans-NN) with C①-C②-C③-N④ close to 0°. The pyridine at the right side maintains the direction with O⑤-C⑥-C⑦-C⑧ close to 180°. (f) The direction of bipyridine flips to 180° at 315.3ps. Both C①-C②-C③-N④ and O⑤-C⑥-C⑦-C⑧ close to 0° indicate the behavior of bipyridine flipped. (g) Both C①-C②-C③-N④ and O⑤-C⑥-C⑦-C⑧ close to 0° mean the bipyridine back to the origin. (h) At 1.0ns, the bipyridine is in the form of trans-NN.

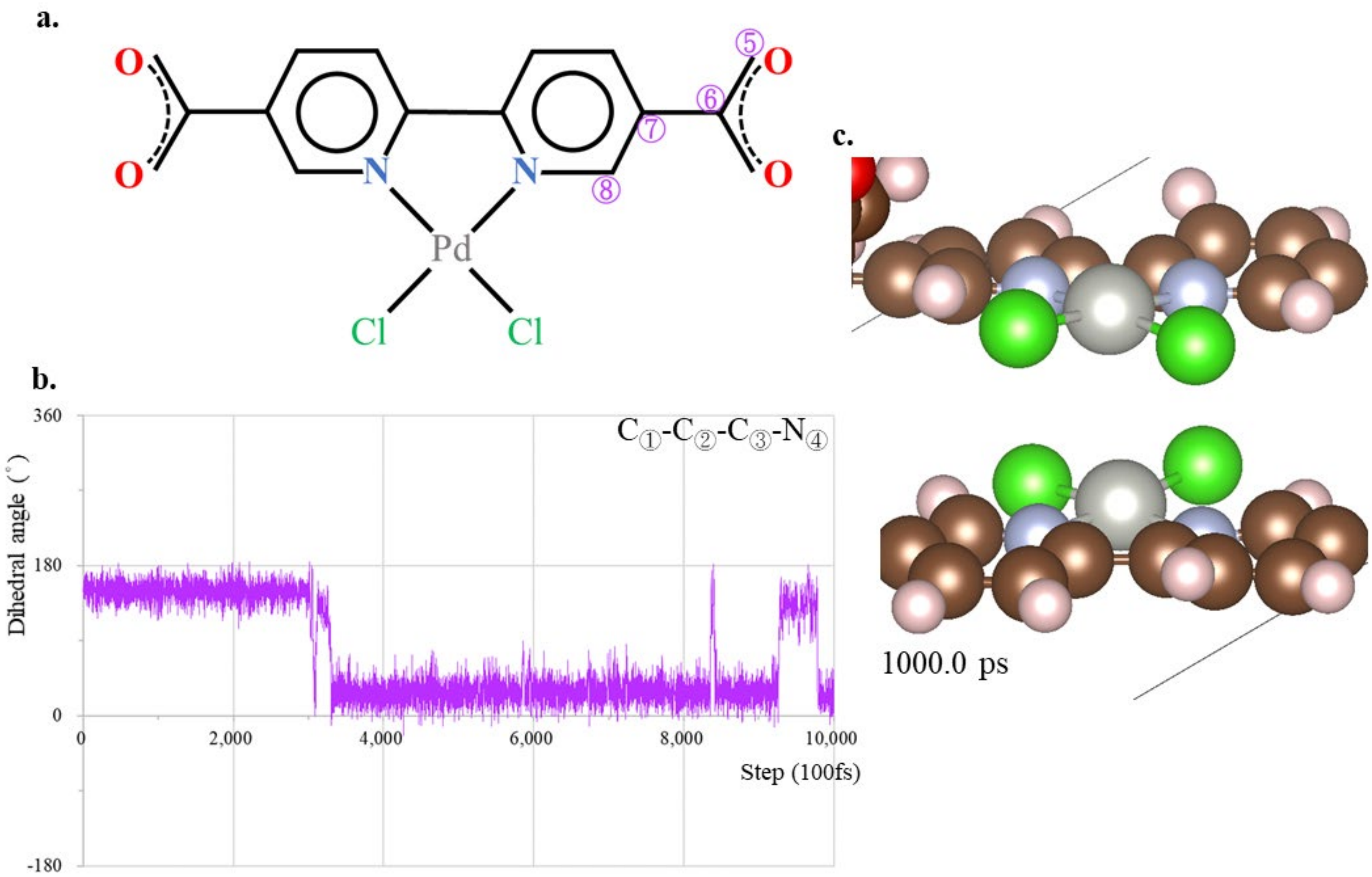

Figure S3. (a) The label shows the definition of dihedral angles, O⑤-C⑥-C⑦-C⑧ in the $LPdCl_2$ model. (b) The purple line shows the change of O⑤-C⑥-C⑦-C⑧ of L from step 0 to 10000 at 1000K. (c) The structure at 1.0ns. The distance between Pd atoms is 3.48 Å.

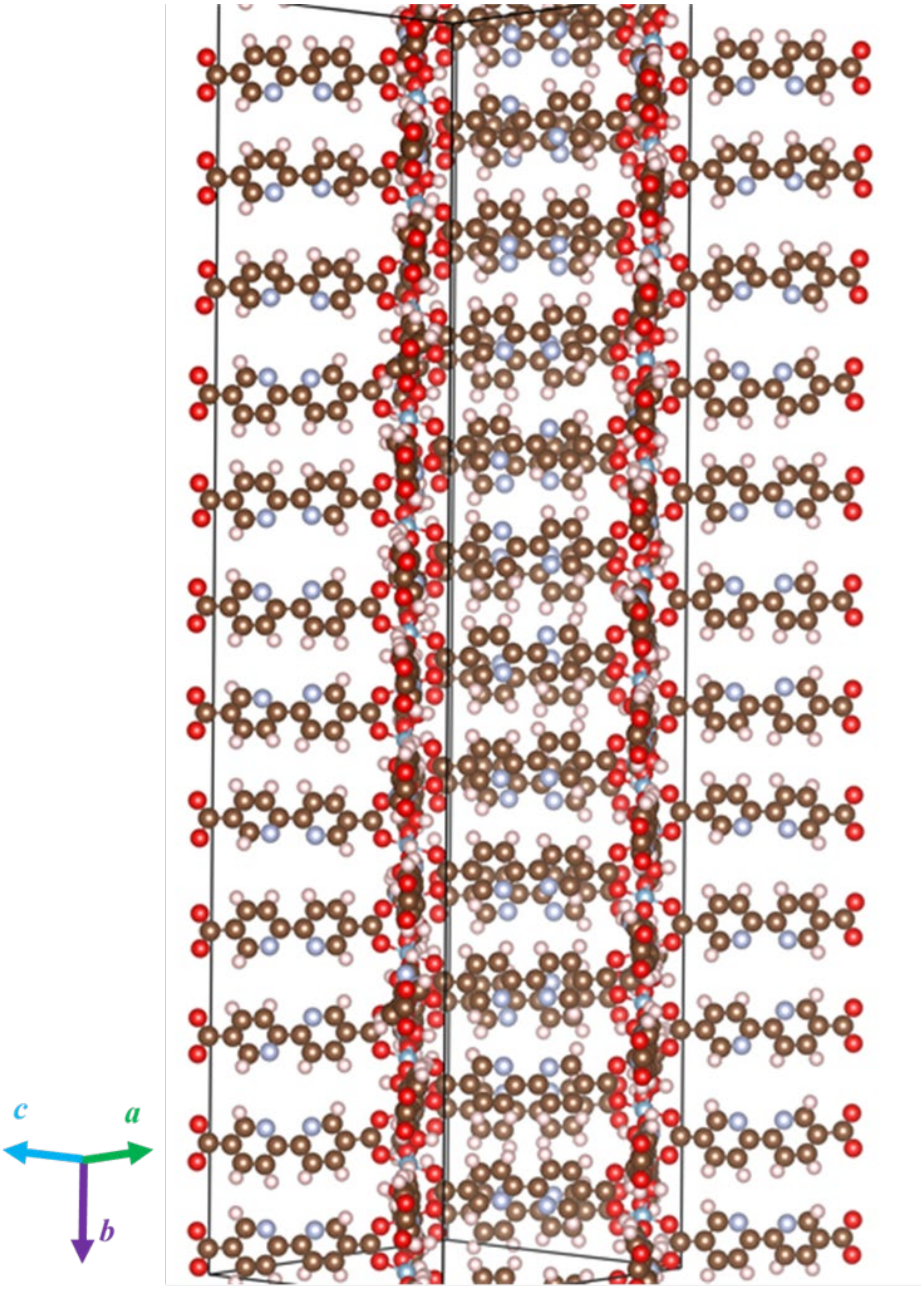

Figure S4. The diversity of linker rotation in the L_6 model at 0.5nm under 1000K.

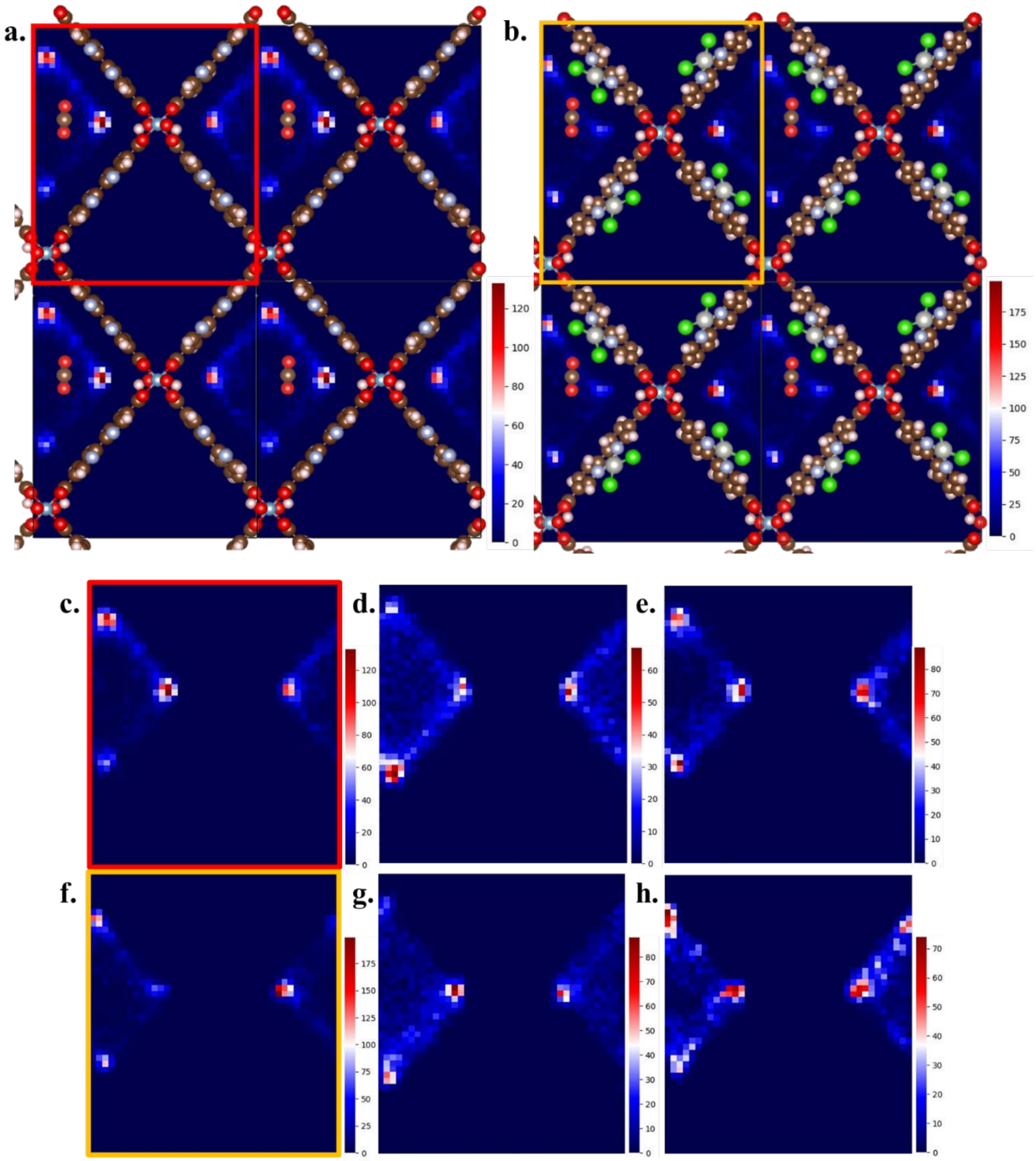

Figure S5. The heat maps show the statistics of the position of the trajectory that a gas molecule in the PBC model of L or $LPdCl_2$ projected on the ac-plane. (a) and (b) as schematics that show the atoms of the L and $LPdCl_2$ and how the heat maps correspond to the cell. Each block in the heat map means a position on the ac-plane with an area of 0.5Å×0.5Å. The red and orange circle point to a unit that could be repeated by the a-axis or the c-axis to form completed pores of the framework. All of the heat maps have a color map on the right with different numbers noted to the frequency for 5000 steps (100fs per step). (c-e) denote the enlarged plots of a (the red rectangle) containing $1CO_2$, 1 H_2, and $1N_2$ using L supercells at 300K, respectively. (f-h) denote the enlarged plots of b (the orange rectangle) containing $1CO_2$, $1H_2$, and $1N_2$ using $LPdCl_2$ supercells at 300K, respectively.

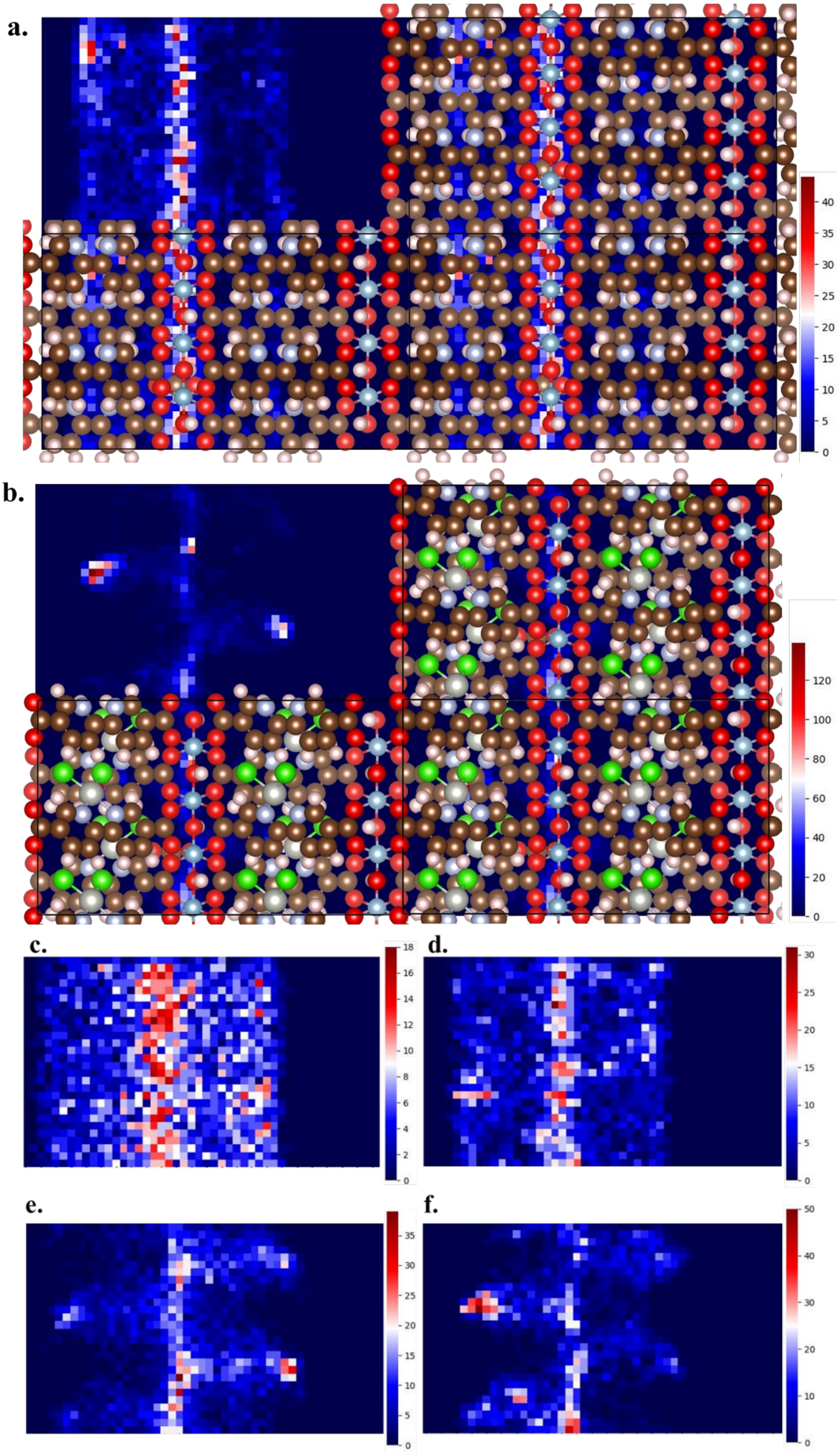
a.
b.
c.
d.
e.
f.

Figure S6. The heat maps show the statistics of the position of the trajectory that a gas molecule in the PBC model of L or $LPdCl_2$ projected on the ab-plane. (a) and (b) as schematics that show the atoms of the L and $LPdCl_2$ and how the heat maps correspond to the cell. Each block in the heat map means a position on the ab-plane with an area of 0.5Å×0.5Å. All of the heat maps have a color map on the right with different numbers noted to the frequency for 5000 steps (100fs per step). (a) is for 1CO_2 in L at 300K. (b) is for 1CO_2 in $LPdCl_2$ at 300K. (c) is for 1 H_2 in L at 300K. (d) is for 1 N_2 in L at 300K. (e) is for 1 H_2 in $LPdCl_2$ at 300K. (f) is for 1 N_2 in $LPdCl_2$ at 300K.

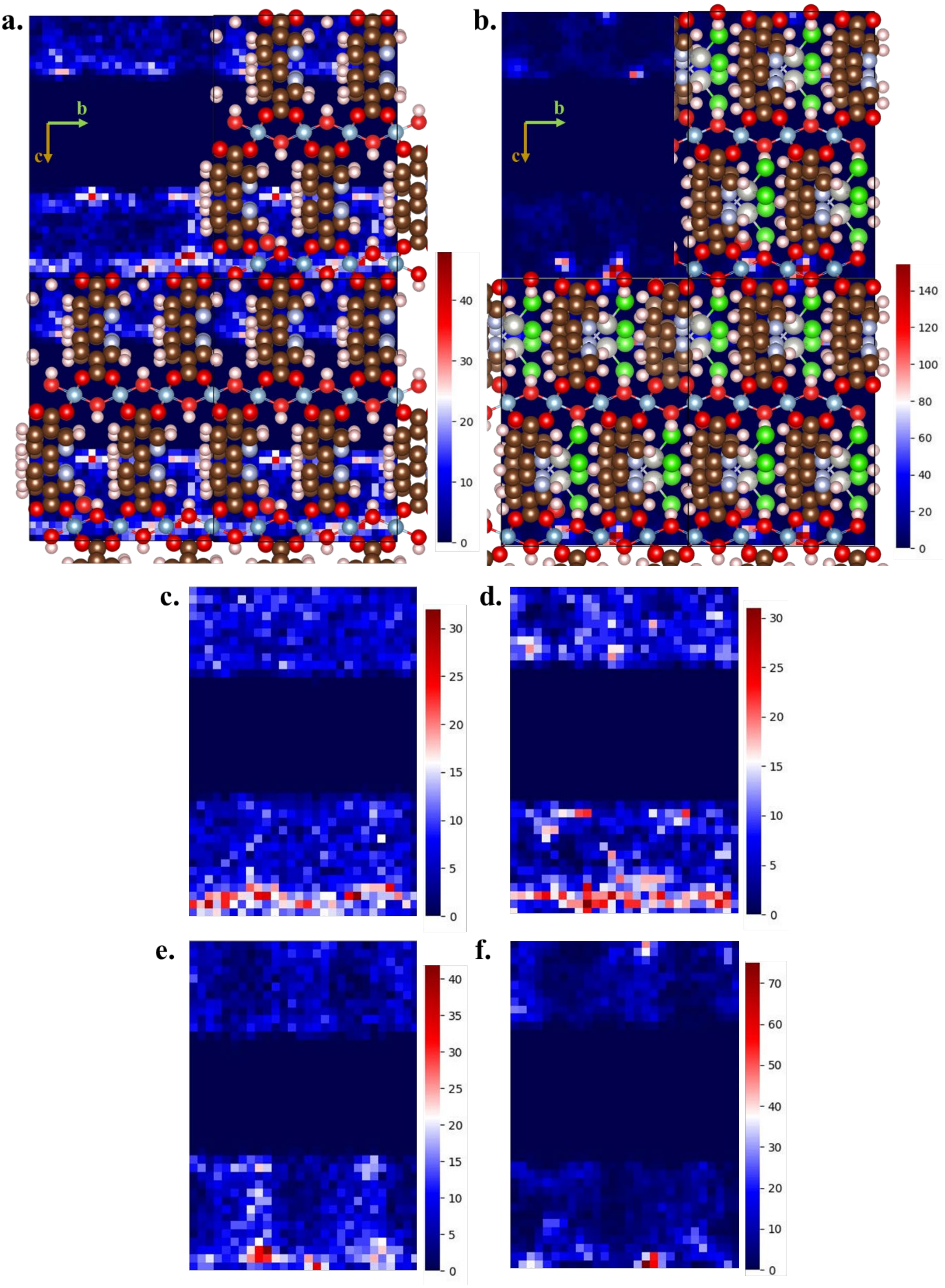
a.
b
c
0
10
20
30
40
b.
b
c
0
20
40
60
80
100
120
140
c.
0
5
10
15
20
25
30
d.
0
5
10
15
20
25
30
e.
0
5
10
15
20
25
30
35
40
f.
0
10
20
30
40
50
60
70

Figure S7. The heat maps show the statistics of the position of the trajectory that a gas molecule in the PBC model of L or $LPdCl_2$ projected on the bc-plane. (a) and (b) as schematics that show the atoms of the L and $LPdCl_2$ and how the heat maps correspond to the cell. Each block in the heat map means a position on the bc-plane with an area of 0.5Å×0.5Å. All of the heat maps have a color map on the right with different numbers noted to the frequency for 5000 steps (100fs per step). (a) is for $1CO_2$ in L at 300K. (b) is for $1CO_2$ in $LPdCl_2$ at 300K. (c) is for 1 H_2 in L at 300K. (d) is for 1 N_2 in L at 300K. (e) is for 1 H_2 in $LPdCl_2$ at 300K. (f) is for 1 N_2 in $LPdCl_2$ at 300K.

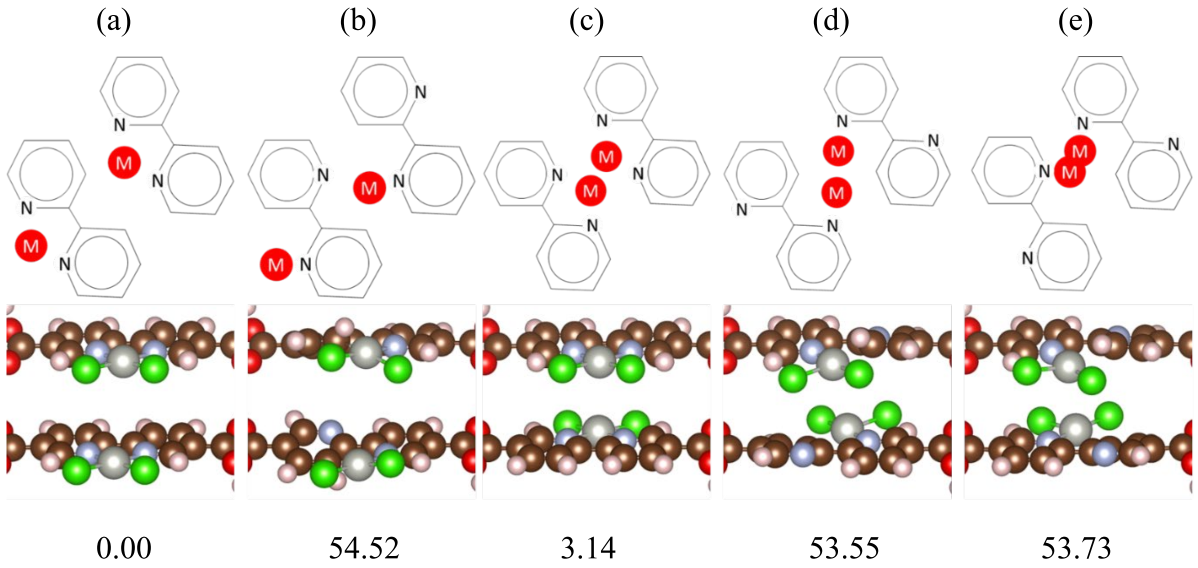

Figure S8. The schematic and DFT-optimized structures of (a) *cis*-unidirectional-a, (b) *trans*-unidirectional-a, (c) *cis*-bidirectional-a, (d) *trans*-unidirectional-b, and (e) *trans*-bidirectional-b orientations for MCl_2-chelation MOF-253 and the corresponding relative energies in kcal/mol.
